# Looking at Fog Computing for E-Health through the Lens of Deployment Challenges and Applications

**DOI:** 10.3390/s20092553

**Published:** 2020-04-30

**Authors:** Pedro H. Vilela, Joel J. P. C. Rodrigues, Rodrigo da R. Righi, Sergei Kozlov, Vinicius F. Rodrigues

**Affiliations:** 1 National Institute of Telecommunications, Santa Rita do Sapucaí, MG 37540-000, Brazil; pedrov@mtel.inatel.br; 2 Federal University of Piauí (UFPI), Teresina, PI 64049-550, Brazil; 3 Instituto de Telecomunicações, 6201-001 Covilhã, Portugal; 4 International Institute of Photonics and Optoinformatics, ITMO University, 197101 St. Petersburg, Russia; kozlov@mail.ifmo.ru; 5 Applied Computing Graduate Program, Universidade do Vale do Rio dos Sinos, Unisinos, São Leopoldo, RS 93022-750, Brazil; rrrighi@unisinos.br (R.d.R.R.); vfrodrigues@unisinos.br (V.F.R.)

**Keywords:** fog computing, cloud computing, e-health, healthcare, Internet of Things

## Abstract

Fog computing is a distributed infrastructure where specific resources are managed at the network border using cloud computing principles and technologies. In contrast to traditional cloud computing, fog computing supports latency-sensitive applications with less energy consumption and a reduced amount of data traffic. A fog device is placed at the network border, allowing data collection and processing to be physically close to their end-users. This characteristic is essential for applications that can benefit from improved latency and response time. In particular, in the e-Health field, many solutions rely on real-time data to monitor environments, patients, and/or medical staff, aiming at improving processes and safety. Therefore, fog computing can play an important role in such environments, providing a low latency infrastructure. The main goal of the current research is to present fog computing strategies focused on electronic-Health (e-Health) applications. To the best of our knowledge, this article is the first to propose a review in the scope of applications and challenges of e-Health fog computing. We introduce some of the available e-Health solutions in the literature that focus on latency, security, privacy, energy efficiency, and resource management techniques. Additionally, we discuss communication protocols and technologies, detailing both in an architectural overview from the edge devices up to the cloud. Differently from traditional cloud computing, the fog concept demonstrates better performance in terms of time-sensitive requirements and network data traffic. Finally, based on the evaluation of the current technologies for e-Health, open research issues and challenges are identified, and further research directions are proposed.

## 1. Introduction

During the past few years, the healthcare industry noticed the potential of how Internet services could help to enhance the patient’s life quality by offering analysis and processing of data in real-time. An efficient model able to provide storage and application processing over the Internet is the concept of cloud computing [1]. This model can be described as a service provided by large data centers that offer part of their infrastructure—both hardware and software—to third parties (corporations and/or individuals) and public organizations. Once these clients adhere to this type of service, they will have computational resources with increasing capacity without the need for significant investments of financial capital for the acquisition, maintenance, and management of such resources [2,3].

Complementing this concept, fog computing expands the services offered by the traditional cloud model to the network boundary [4]. Fog computing has as its main characteristics low latency, the greater geographic distribution of data, mobility, large numbers of nodes in the network, predominantly wireless access, execution of real-time applications, and device heterogeneity [4,5,6]. Its purpose is to enhance efficiency, performance, and reduce the volume of data sent to the cloud for processing, analysis, and storage. Nonetheless, the data collected by the sensors is gathered, processed, and stored in a temporary database instead of handing it to the cloud, thus avoiding round-trip delays and network traffic. This feature is particularly crucial for electronic-Health (e-Health) applications that transmit data over the Internet for remote real-time processing, such as remote ECG monitoring [7]. Such applications aim to monitor patients and/or processes actively [8], thus producing valuable information for decision making.

One of the reasons for the emergence and implementation of fog computing was the need to create a platform that would support the recent paradigm inherited from ubiquitous computing, the Internet of Things (IoT) [9], where any object may act as a sensor node and offer a particular service, such as data processing. In this context, CISCO [10] predicts that in 2020 the volume of data generated by IoT devices may reach six hundred zeta bytes per year. This scenario implies significant challenges to how data is exchanged among devices and the cloud, due to the high demand for bandwidth and network latency. In the context of e-Health, such issues pose some challenges regarding how to handle an increasing amount of data to maintain low latency for real-time applications. Although cloud and fog computing offer similar services, there are differences when considering the context of fog computing. With the massive amount of data arising from the end-devices, using remote cloud networks to transport data may become impractical or resource-prohibitive [11,12].

E-Health applications are a group of software and services focused on the acquisition and transmission of medical information used to deliver healthcare services [13,14]. Typically, these applications require higher levels of security and quality of service (QoS) from the system infrastructure. Currently, a few studies focus on presenting literature surveys and reviews in the scope of fog computing and e-Health applications [15,16,17,18,19,20]. However, they focus mostly either on presenting characteristics of strategies and challenges that might be a target for future research, or specific health scenarios, such as smart homes [20]. These studies do not describe a comprehensive taxonomy regarding the main characteristics of e-Health applications employing fog computing, and also do not perform an analysis considering a comparison between cloud and fog environments. With that in mind, the main objective of this paper is to update the current state-of-the-art on fog computing, focusing our discussions and contributions on the application requirements, their challenges, and open gaps still existing in the literature.

In this context, the current research presents details of fog computing and e-Health applications, analyzing the main strategies present in the literature. Based on these papers, we propose a taxonomy for the joint combination of e-Health and fog computing, thus defining the characteristics of the main applications encountered in fog computing deployments. The most relevant contributions of this study are listed as follows:

(i) An analysis of how e-Health applications benefit from the fog computing architecture in terms of deployment, communication protocols, data security, and infrastructure details;
(ii) A mapping regarding the focus of e-Health systems employing the concept of fog computing from the point of view of application requirements and main tasks;
(iii) A definition of the concerns and challenges regarding the joint combination of e-Health and fog computing, also giving directions for further research and developments.

The remainder of this paper is organized as follows. Section 2 introduces relevant concepts addressed in the paper. Section 3 presents a short overview of research initiatives related to the current research. Section 4 describes the method followed to perform the study. Section 5 presents the results of the survey; in particular, Section 5.1 describes the main architecture used in e-Health applications and identifies the most relevant fog computing features, Section 5.2 outlines the most relevant e-Health scenarios and applications under use, and Section 5.3 discusses lessons that emerged during this study. Finally, Section 6 presents the final considerations, again highlighting our contributions.

## 2. Background

This section presents the current state of e-Health applications regarding the fog computing concept, describes the practical approaches already under use and explains the need for a new architectural model to deal with the massive amount of generated data, especially by IoT smart devices. The focus of this section is to depict the healthcare scenario involving the IoT environment and outline its major application scenarios. It also presents the definition of some computing paradigms, such as cloud and fog computing, and compares them in terms of their computational capacity and critical network metrics.

### 2.1. E-Health Scenarios

Many countries are facing a considerable challenge to manage a rapidly growing aging population and the increase in chronic diseases [21,22]. The demand for medical care has risen in recent years due to the popularization of IoT smart devices, which opens a field of study for new models of delivering medical services that improve the way health information is handled. Currently, the conventional method consists of patients visiting their doctors only when they fall sick. To check their health conditions, they frequently visit hospitals or clinics to meet their physicians. As a consequence, most of the time, health parameters are manually monitored and transferred to healthcare systems, which leads to inefficient use of resources, and sometimes to higher costs. With the employment of e-Health smart sensors and medical devices, many manual tasks could be released from caregivers, since patients’ conditions can be automatically monitored and analyzed remotely [23]. This new technique may revolutionize the way diagnostics and treatments are performed. Another important fact is the possibility to use healthcare systems for patients’ health monitoring to keep them out of hospitals and, thus, improve hospital resource management. Furthermore, employing IoT devices for monitoring patients remotely enables them to receive medical care ubiquitously [24]. By analyzing the e-Health scenarios, we can affirm that each one can be enclosed in at least one of the goals: (i) mobility support [25]; (ii) ambient assisted living (AAL) [26]; and (iii) in-hospital treatment [8].

For the first group, with the popularization of the Internet in mobile devices, a new paradigm of e-Health has emerged: mobile-Health (m-Health) [25]. Although there is no standard definition for this concept yet, according to the World Health Organization (WHO) [27], m-Health may be understood as the offering of medical services through mobile devices such as mobile phones, sensors, and other wearable devices. This scenario also encompasses the connectivity in an ambulance or any medical air transport. In turn, the concept of AAL, as stated in [26], intends to link the usage of biomedical system monitoring with other environmental smart device sensors to provide more efficient assistance to people who live alone or have a particular disability/chronic disease. For that means, AAL performs a fundamental role in healthcare by detecting possible accidents or indicating evidence of abnormality, which helps people with their daily routine and, at the same time, helps to reduce government spending on elderly healthcare. Finally, in-hospital treatment encompasses all medical equipment used to monitor and analyze vital sign parameters from patients within hospital care [8]. Compared to the scenarios mentioned above, the devices in this context are more complex and are usually owned and maintained by the hospital itself.

### 2.2. Cloud Computing

Cloud computing, as stated in [28], is a prominent concept of delivering computational and storage as a service to a shared pool of selectable resources. Users can request and/or decline any computational processing or storage they may require automatically. Differently from traditional services, this approach enables the development of elastic applications that are charged accordingly to their use. As the authors in [29] mention, one of the attributes of cloud computing applied to healthcare is the possibility to consume resources from the cloud whenever needed and pay only for the used resources. Another great advantage is the capability of sharing information among health professionals, caregivers, and patients in a more structured way, reducing the risks of losing documents such as exams and medical records. However, the majority of the cloud data centers are geographically centralized and located far from its end-users. In that event, real-time applications sensitive to delays suffer from some issues, such as high round-trip delay and network congestion.

### 2.3. Fog Computing

With the arrival of the IoT, much has been planned on how to execute all information processing brought about by smart devices correctly, and that is precisely what the concept of fog computing tries to solve. Its purpose is to make the processing of generated data occur directly at the device, or next to it, at the network boundary, in more powerful equipment, with no need to send it to the cloud [30]. The term “fog computing” was first introduced by CISCO, and it is defined as an architecture that extends the computational and storage capacity of the cloud to the edge of the network [29]. In other words, it is a cloud infrastructure closer to its end-users. Consequently, it allows data to be collected and processed locally, reducing network latency as well as bandwidth usage. The main benefits brought by the fog computing paradigm found in the literature are: (i) reduced latency [31,32]; (ii) enhanced privacy [33]; (iii) lower need of bandwidth [10]; (iv) dependability [34]; (v) energy efficiency [35]; and (vi) data security.

First, handling data at the network’s border reduces the latency when compared to other cloud-based architectures, since the physical distance is shorter. Therefore, potential data center delays may be avoided. Another advantage brought by the fog computing concept is the possibility to move computation-intensive tasks from devices with limited resources to a more powerful node [31,32]. Second, differently from the cloud model, the privacy of user data may be enhanced once the fog approach enables the analysis and processing of data on a local gateway, instead of sending the information to the cloud [33]. In other words, the number of hops that the user’s data are transmitted over the network is smaller, reducing the data’s exposure to external routers and networks. Third, since the fog model enables the data to be collected and processed closer to end-users, the volume of data transferred to the cloud is reduced, avoiding network traffic expenditures. This is possible because only a small part of the data is sent to a remote cloud data center for storage, whereas the rest of the data is analyzed and processed at the user location [10]. Fourth, the fog paradigm can improve the system’s dependability by sharing the same functionality among the many different fog nodes. Thus, it enhances data redundancy. Further, since the computational resources are placed closer to end-users, the system may be less dependent on network connection availability [34]. Finally, energy is a primordial item that must be carefully analyzed in the IoT environment. When talking about sensor devices, reducing energy consumption is very important, since most of the sensors are battery-driven. In such cases, the overall energy efficiency can be improved by employing fog nodes acting as gateways. Such gateways can handle requests or update processes while the sensor is in a sleep state. Whenever it wakes up, it takes control of the whole application. Additionally, tasks requiring more intensive power processing can be offloaded from energy-constrained devices to nearby gateways [35].

## 3. Related Work and Motivation

This section presents current studies that present surveys or review articles in the scope of fog computing and healthcare in the last few years. More precisely, this section aims to demonstrate related research with this article in a way that it is possible to demonstrate its contributions compared to current research. In the fog and health scope, a few articles propose systematic literature reviews employing different methodologies [15,16,17,18,20,36]. In [18], the authors present a literature review on pervasive health applications, focusing on identifying characteristics of such applications that might benefit from fog computing environments. The authors discuss which computational tasks from e-Health sensors can be moved to fog computing infrastructures and where these tasks can be executed. Combined with this, the authors examine the trade-off of placing the identified computational tasks in the network. The study consists of a systematic literature review based on three research questions. Although the article focuses specifically on fog computing, their inclusion criteria consider a broad set of terms from the wireless sensor networks field. However, this broad set of articles is used to extract possible environments in which fog computing might play an important role. The authors identified in the literature five deployment scenarios that can benefit from fog computing: mobile, home, hospital, non-hospital, and transport. With a different scope, in [36], the authors perform a systematic literature review in the context of resource management in fog environments answering six research questions. Their inclusion criteria consider articles a set of words related to resource management plus fog or edge computing. They reviewed a group of 100 articles with publishing years ranging from 2014 to 2018. One of the main outcomes of their study regards a taxonomy of resource management approaches with the respective article for each topic.

The authors in [17] performed a literature review to summarize the main domains and issues related to fog computing and healthcare. Their primary focus was to provide an overview of fog issues tackled by the literature versus the application domain. Their method consisted of employing a systematic literature review to answer three research questions in the specific field of fog-health: the statistical publication trends according to publisher and date; the application domains; and the most discussed issues. For the first issue, they organized the articles to demonstrate the research interest in the subject in the previous few years. Their main contribution relies on an evaluation of the relationship between domains and issues in this specific research field. From their literature corpus, they found that data analysis and response time issues for remote health monitoring are the main studied characteristics. By analyzing the literature, the authors demonstrated that many applications present strategies regarding time-delay in the health scenario due to its critical importance for the treatment of patients. Silva and Júnior [16] presented a literature review on fog computing for healthcare, focusing on the state-of-the-art and challenges in this field. The paper seeks to answer what are the types of applications used, the aimed-at diseases, the characteristics of the fog solutions, the reasons for each research, and what are the main challenges. Among the authors’ main findings, the most important regards the lack of a well-defined fog architecture in the context of healthcare. The search strategy employed by the authors considered studies that use fog computing in healthcare scenarios to answer six research questions. Although the authors did not define specific years for the studied articles, their strategy resulted in a short year range (2015–2018) that considers less than four years since the research was done in early 2018. From the resulting corpus, for each research question, the authors classified the articles according to their focus.

In [15], the authors presented a systematic literature review of healthcare IoT applications employing fog computing with shared resources. The article performed a systematic literature review with main inclusion criteria that encapsulate cloud computing, fog computing, and edge computing. Their focus was to present the current literature research status in fog computing in the healthcare applications field, show the performance evaluation objectives of the state-of-the-art, define the main methods they employ, and find some future directions in this research topic. From the resulting corpus, the authors focused specifically on technical details from strategies of fog computing in healthcare IoT systems. This literature strategy resulted in a broad set of articles that the authors classified within their three-category taxonomy: methods, system development, and review and survey. According to the authors, these categories were derived from previous studies and aimed to cover articles that focus on models, implemented architectures, and literature reviews. In [20], the authors presented a literature review focused on fog computing for smart home scenarios. The authors’ strategy consisted of a systematic literature review that seeks to answer six research questions regarding fog-based smart home applications. To do so, their inclusion criteria considered the terms “fog” and “smart” combined to a set of words referring to building and homes. Based on the reviewed articles, the authors proposed a taxonomy categorizing the solutions that are focused on resource management or service management. Differently from the other studies, In [19], the authors did not employ a systematic literature review in their research process. Instead, they discussed characteristics of edge and fog computing environments and how they can be applied for healthcare applications. The authors aimed at describing the principal concepts of edge and fog computing that are important in the context of healthcare. Their main contributions focus on presenting strategies for how to combine such technologies to distribute computing tasks that are currently performed in the cloud computing layer. The authors presented an analysis of health IoT applications integrating the fog computing layer between the hospital infrastructure and the cloud environment. This layer allows applications to respond quickly in case of a medical crisis, since the fog processing capabilities are closer than the cloud.

Table 1 summarizes the six related works presented in this section. Half of the articles focus only on fog computing as technology, while the other half also include at least one more technology in their evaluation. Considering if they present a taxonomy in their research or not, the last three articles propose a taxonomy with their findings [15,20,36]. On the one hand, the authors in [15] presented a taxonomy dividing the strategies into three categories in which they classified if the articles present methods or systems, or perform literature reviews. On the other hand, in [20], the authors built a taxonomy dividing the articles into two categories that classify the articles according to whether they are service-based or resource-based. Besides them, the authors in [36] presented a taxonomy focusing specifically on resource management approaches and the respective articles tackling each category. The current related work demonstrates that the studies focus mainly on presenting fog computing as a new layer between the end-users and cloud infrastructures. Additionally, the studies present some contributions by defining the main characteristics of solutions employing fog computing to healthcare scenarios. Considering all the studies presented in this section, they fall into at least one of the following affirmations: (i) papers do not describe a comprehensive taxonomy regarding the main characteristics of healthcare applications employing fog computing; and (ii) articles do not perform an analysis considering a comparison between cloud and fog environments. The next sections of the current article focus on exploring these issues.

## 4. Literature Review Methodology

This study presents a literature review based on the principles of systematic literature reviews [37] in order to make it reproducible and achieve high-quality results. This section outlines the research methodology, presenting the strategy used for the collection and selection of the most appropriate contributions and corresponding papers. The goal of this research is to summarize and update the current state-of-the-art of e-Health applications employing the concept of fog computing and present its major characteristics in terms of its computing tasks. Therefore, Table 2 defines a set of research questions (RQ) that guide the review process of this study. The importance of the research questions is to provide a better understanding of the impact of fog computing on e-Health applications. Furthermore, it helps to identify the major characteristics of systems that have already deployed the fog concept concerning architectural models, network metrics for performance evaluation, security issues, and so on.

**Figure 1:**
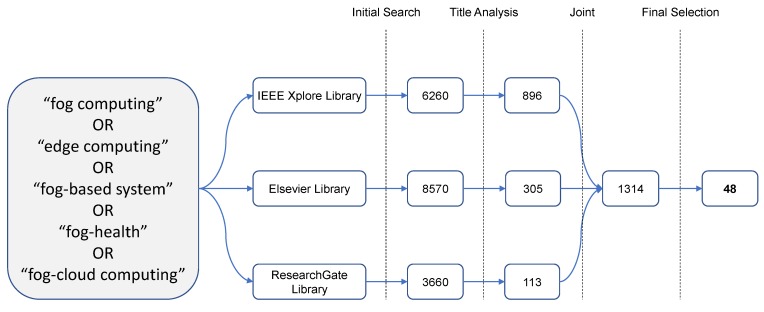
Summary of the systematic literature review process.

The literature selection was based on journal and conference papers with the aid of electronic database resources. The following databases were queried: the Institute of Electrical and Electronics Engineers (IEEE) IEEExplore digital library (https://ieeexplore.ieee.org/); the Elsevier journal directory (https://www.elsevier.com/); and ResearchGate social networking (https://www.researchgate.net/). The inclusion criteria consisted of querying these databases with different keywords to collect the raw literature corpus. In each database, the following set of keywords was applied in the search string: “fog computing”, “edge computing”, “fog-based system”, “fog-health”, and “fog-cloud computing”. Figure 1 presents the whole process of collecting and selecting the candidate articles for the review. The resulting raw corpus of articles contained 18,490 articles, to which a title analysis was applied as a first filter to decrease the number of candidates. This process resulted in 1314 articles that were joined for the last filter phase, which consisted of exclusion criteria.

The exclusion criteria process was employed through a first reading phase in order to filter the most relevant research to be included in this study. The exclusion consisted of the following removal steps: (i) removal of papers written in languages other than English; (ii) removal of redundant and/or unwanted papers that are not related to fog computing or e-Health applications; (iii) removal of books, manuals, theses, and papers not related to fog computing; and (iv) removal of papers mentioning fog computing but not applied to healthcare. Finally, at the end of the process, 48 articles were selected to be reviewed and included in this study. This resulting corpus of studies was carefully analyzed to identify their main aspects. The next three sections are organized in a way to reflect the three research questions, and the articles are reviewed in them aiming at answering the RQs presented in Table 2.

## 5. Results

This section presents the answers for the three questions detailed in Table 2.

### 5.1. RQ1: How Do E-Health Applications Benefit from the Fog Computing Architecture?

To answer that question, it is first necessary to understand the main characteristics of fog computing deployments present in the literature. Therefore, Section 5.1.1, Section 5.1.2, Section 5.1.3 and Section 5.1.4 present the main characteristics of such deployments present in the literature regarding architectural model, communication, infrastructure, and security, respectively. Then, Section 5.1.5 discusses the most important details and presents a resulting taxonomy that depicts the main findings regarding fog computing in the scope of health applications.

#### 5.1.1. Architecture Model

**Figure 2:**
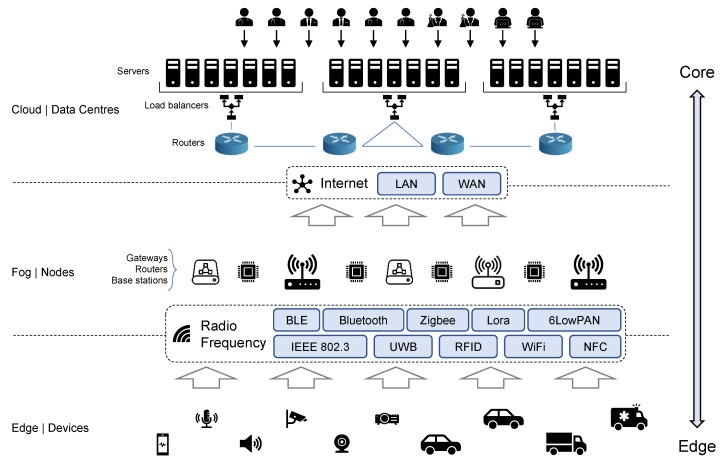
Illustration of a basic three-tier fog architecture presenting some sensors, devices, and communication protocols present in the model.

As a new approach for computation, fog computing supplements the classic cloud computing and its services closer to its end-users. For this purpose, it is capable of providing computation and storage resources in a decentralized model. The main concept of fog computing architecture is a promising subject in the telecommunication research field. Recently, many architectures for fog computing have been presented, where the three-tier architecture is considered to be the most predominant structure [4]. The basic architecture deployed in the fog computing paradigm is illustrated in Figure 2 and is composed of the following layers: edge, fog, and cloud. The edge (device) layer is the closest layer to end-users/devices. It consists of several devices, such as sensors and mobile phones. The devices in this layer are responsible for collecting data from physical objects and sending them to the upper layer through short-range radio frequency technologies. Located at the edge of the network, the fog layer is comprised of network devices, such as routers, gateways, and base stations, among several others. Such devices/nodes are responsible for tasks such as scheduling, storing, and managing distributed computation. The third layer is the tier with sufficient storage and computational resources, responsible for extensive data analysis and permanent storage. Different from traditional cloud architectures, this layer accesses the cloud core network in a periodical and controlled way, improving the utilization of available resources [38].

In [39], the authors present an architectural design for IoT systems. In this design, the cloud works as an extension of the fog layer in an assistive way. The fog gateway is placed between the cloud and the user devices, such as sensors and actuators, to meet network requirements as well as to manage and provide resources to several distributed fog nodes. According to [40], offering computation and storage at the edge of the network also helps to reduce bandwidth usage and mitigates security and privacy concerns. Several approaches place their computation task on a single node of a personal area network (PAN) or local area network (LAN) [41,42]. The data collected at this level are processed and sent to the upper level and, sometimes, to the cloud. Other approaches employ two or more fog nodes linked between the device sensing and the cloud access points [43,44].

#### 5.1.2. Communication Protocols

IoT protocols are very relevant when performance and energy efficiency are required. In the study presented in [34], a detailed research was carried out for evaluating some of the most important protocols applied to the e-Health system, in terms of performance and energy consumption. The main protocols used in e-Health applications can be classified into three groups: (i) constrained application protocol (CoAP); (ii) Internet protocol version 6 over low power wireless personal area network (6LoWPAN); and (iii) message queuing telemetry transport (MQTT). CoAP is an Internet application protocol defined by the RFC 7252 [45] that is designed for resource-constrained sensor devices [46]. It is most explored in wireless sensor networks (WSN) in which devices have energy constraints and need a lightweight communication protocol to perform their operations. It implements methods similar to hypertext transfer protocol (HTTP), where data is encoded in a simple binary format, often based on JavaScript object notation (JSON). CoAP uses the representational state transfer (REST) style to make the resources available over the user datagram protocol (UDP) [42,46,47,48]. One of the the main ideas behind CoAP is to allow machine-to-machine (M2M) communication while keeping the message overhead small to avoid packet fragmentation, thus increasing the packet probability delivery [45]. WSNs are typically deployed in e-Health systems for data collection. By this means, according to [47], the utilization of lightweight protocols helps to speed up the response time once the amount of data exchanged from the application and the back-end system is smaller.

6LoWPAN is a protocol that allows data transmission between low power devices over IEEE 802.15.4 networks [49]. The IEEE 802.15.4 is a communication standard that aims devices with low-data-rate and low-power capabilities, allowing them short-range radio frequency transmissions in low-rate wireless personal area networks (WPANs) [50]. 6LoWPAN is designed on top of this standard to provide such devices with the ability to use the Internet protocol capabilities. Its specifications can be found in different RFC definitions, since each one deals with specific subjects: RFC 4919 [51], RFC 4944 [52], RFC 6282 [53], RFC 6775 [54], and RFC 7668 [55].

Finally, MQTT is a very common protocol, defined by the ISO/IEC 20922 [56], present in IoT environments [46]. Its main goal is to provide M2M communication through the exchange of bi-directional messages so that remote nodes can communicate using the MQTT infrastructure. It is an extremely lightweight and simple protocol designed to operate in hardware-constrained devices and offer low bandwidth consumption. MQTT architecture is based on transmission control protocol/internet protocol (TCP/IP), and its messages are exchanged through the publish/subscribe paradigm. Such a paradigm is composed of a component called a broker, which is responsible for receiving, queuing, and dispatching messages from publishers to subscribers [41,46,57].

#### 5.1.3. Infrastructure Technologies

Infrastructure details involve both communication and hardware technologies used to set up fog environments in the health scope. E-Health systems typically employ a combination of networks to interconnect medical devices to the cloud. Among several networks, some commonly appear in the literature: wireless body area networks (WBANs), WPANs, LANs, and wide area networks (WANs). Using a particular technology for radio frequency, WBANs and WPANs can be seen as networks that enable the connection of devices acquiring personal information close to the patients. While WBAN refers to wearable sensors attached to patients [58], WPANs offer a higher layer on top of WBAN, allowing sensors and devices to communicate among themselves and to access local networks [59]. On the other hand, LAN and WAN are the already-spread networks for local and long-range communication. In a mobile scenario, the majority of sensors are connected to an Internet access point via Wi-Fi communication [60,61]. Another way to connect sensors is through wireless sensor and actuator networks (WSANs), which consist of a group of sensors and actuators wirelessly connected that perform distributed sensing and actuating tasks. A fog-based gateway architecture for WSAN is presented in [62], where the vitalized model is a joining of a gateway and a micro server connected via Ethernet. The communication of the interface between the gateway nodes may be wired (e.g., Ethernet) or wireless, such as third generation (3G), and long term evolution (LTE).

Regarding hardware technologies, there are several development platforms found in the literature for both research and commercial purposes. Such platforms are based on mobile computer boards that allow programmers to develop and quickly prototype their systems. The Arduino is one of the most popular platforms used in many applications due to its ease of programming and its low cost. It is a printed circuit board consisting of ATMEL microcontroller input/output circuits, which can be easily connected to a computer and programmed in the Assembly, C, and C++ languages [63]. Intel Galileo is a 32-bit open-source development board specially designed for IoT applications. It is compatible with Arduino and its respective shields. The board supports blacktooth, Wi-Fi, radio frequency, and Ethernet communication. Additionally, Intel Galileo supports a variety of sensors, such as temperature, electrocardiography, and oxygen concentration, among many others [64]. Another development board is the Raspberry Pi, which is a complete plug-and-play computer with an integrated processor and random access memory (RAM) chips used for IoT development. Despite its higher cost compared to the others, the small device is trendy because of its massive number of open-source packages and libraries, which can be easily implemented using Python, for instance. Besides, the board can be equipped with Ethernet, Wi-Fi, or blacktooth interfaces [65]. Finally, Pandaboard is based on a small advanced RISC machine (ARM) computer from Texas Instruments. It is commonly used for research development purposes because of its minimal energy consumption as well as its low cost. It works with several network standards, such as Wi-Fi, blacktooth, and Ethernet [66].

The literature does not only include physical development platforms but also simulation tools that permit researches to evaluate the effect of their software solutions. An evaluation environment for real-time applications employing fog computing is necessary to enhance the innovation and development of new technologies. As testbeds in the real world are, most of the time, very expensive, the development of software for simulations proves to be an efficient tool to address these problems. Focusing on cloud computing environments, the authors in [67] proposed a framework called *cloudSim* for the simulation of public/private cloud environments. The platform allows the user to model its cloud environment to perform several tests and evaluate its performance before deploying a production environment. In the fog scenario, in [68] the authors proposed a platform called *ifogSim*, which is a toolkit for the modeling and simulation of resource management written in JAVA that intends to minimize latency, energy consumption, bandwidth usage and operational costs. Currently available in the cloud, *ifogSim* provides a simulation environment in which it is possible to model and test massive scenarios of IoT and fog environments.

#### 5.1.4. Security Issues

IoT applications in healthcare must be able to keep medical data private and safe from unauthorized access. Any part of the system exposed to a hacker or malicious software may cause critical consequences. Many researchers are concerned about how to solve security issues in IoT systems since security cannot be 100% guaranteed. Thus, health professionals and caregivers must define an acceptable risk limit of IoT applications. An evaluation of the kind of data that is being processed is a critical task. Depending on its purpose, failures may have a huge impact on the patient’s life. Hence, it is an essential requirement that needs to be considered to keep the system resilient against security threats. The literature presents some studies that address different security aspects: (i) authentication [69]; (ii) privacy [43,70,71,72,73]; and (iii) data encryption [9]. Because of the huge amount of devices within a fog environment, user authentication plays an important role in keeping the system safe from unwanted access to healthcare services. On the other hand, privacy is one attribute of extreme importance in e-Health applications. Patients expect their private information to be kept confidential. However, IoT health systems must allow information sharing, which is necessary for delivering high-quality care and at the same time ensure its privacy. Policies and techniques must be improved to share health data with authorized users only. In addition to the first two security issues, data encryption is also necessary, since it does not matter for the infrastructure. Patients’ data are very sensitive to leaks. Although fog computing normally processes data locally, on some occasions data must be forwarded to the cloud. Therefore, fog nodes must guarantee that those data are adequately encrypted before sending them to remote environments.

In [69], the authors propose a security policy management approach to address the major challenges that are important to keep data sharing and its collaboration secure within a fog computing environment. Furthermore, the authors in [43] reinforce that risk management with all stakeholders is key for achieving optimal safety and performance of medical devices. Currently, there are a few legal commitments to preserve private data in medical applications employing IoT smart devices. In [70,71], some of these legal obligations are presented. In [72], the authors present an architecture for autonomic security management that can assess risks in healthcare information systems applying a cost-efficient self-protecting approach with little or no human intervention at all. This framework also offers prevention mechanisms for monitoring and management solutions helping decision making actions based on security issues. The authors in [73] came up with a framework concerning how to keep private health information safe from eavesdropping or malicious manipulation. Based on this framework, the authors developed a medical expert system to tackle low effectiveness due to manual operations and privacy breaches caused by the participation of doctors in the medical information process. With encryption in mind, in [9] an encryption layer is implemented in the proposed fog architecture.

#### 5.1.5. Discussion

In the field of healthcare, one of the most critical metrics to handle critical situations is time. Considering the health status of patients, quickly diagnosing anomalies in health parameters may improve the physicians’ time response to it and, consequently, save lives. One of the main characteristics of fog computing is the new layer between health sensors and the cloud data centers that process data [4]. This new layer is designed to be physically close to the sensors, and it also provides processing of data for quick responses. From the network point of view, this infrastructure closer to sensors allows data communication over the network to have lower latency levels. Consequently, by inserting this new feature to the e-Health application infrastructure, it is possible to decrease the time response of the system and gain time, which, as mentioned before, is important with regards to patients’ health. Therefore, the main contribution fog computing provides for e-Health applications is this improved network infrastructure for rapid data transfer and processing.

**Figure 3:**
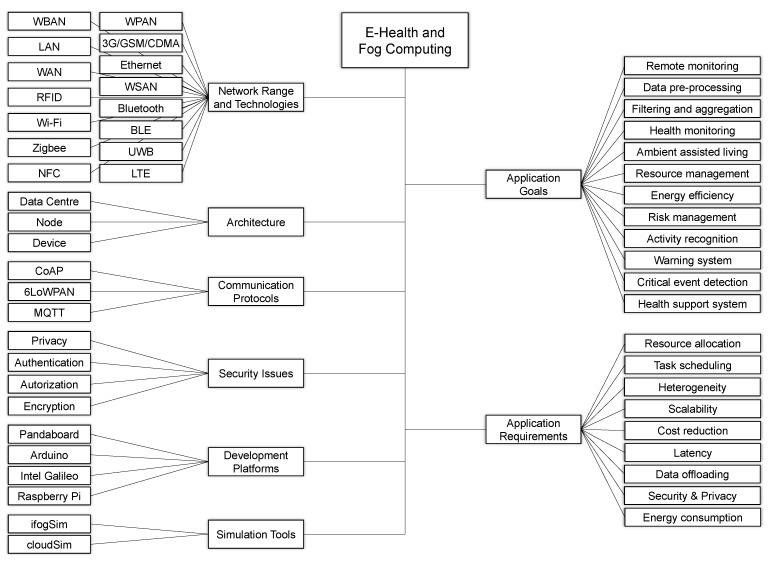
Proposal of a fog computing taxonomy in the scope of e-Health applications.

Figure 3 depicts the proposed taxonomy showing the primary architecture model employed in the fog computing concept. It also presents the most used light-weighted protocols and platforms used in the development of medical applications in the current literature. Security and privacy issues are presented in terms of how the fog concept can enhance the privacy breaches of health information in the current model due to the vast exchange of data among IoT sensors and the cloud.

### 5.2. RQ2: What Is the Focus of E-Health Systems Employing the Concept of Fog Computing?

To answer the above question, this section describes the e-Health applications published in the current literature related to the fog computing paradigm. Such applications are classified in groups accordingly to their main computing task: data collection, data processing, critical data analysis, and real-time feedback. The next four sections individually describe articles that fall into these classifications. Then, Section 5.2.5 discusses the main findings, also presenting a comparison between all article reviews for each class.

#### 5.2.1. Data Collection

As more and more IoT devices are connected to the Internet, the huge volume of data generated will require real-time responses. Therefore, this amount of data implies high bandwidth costs. If false data is introduced in these IoT devices, besides compromising the accuracy of the data, it may increase the use of communication resources. Some studies focus on data collection strategies in order to decrease bandwidth needs and improve response time. Among different strategies, these studies fall in at least one of the following: (i) remote monitoring [74,75]; (ii) pre-processing [76]; (iii) compression [77]; and (iv) filtering and aggregation [76,77,78]. Remote monitoring systems focus on tracking the health conditions of patients outside health environments. In [74], the authors propose the eWall project, which is a system to monitor and supervise patients with mild dementia and chronic obstructive pulmonary disease (COPD) at home. The fog concept applied to this project aims at speeding up data processing in real-time for emergencies. Additionally, in the remote monitoring scope, the authors in [75] proposed an evaluation of a fog-based smart monitoring system using long-range (LoRa) radio communication in remote locations where there is no Internet connectivity. The suggested system helps minimize the power consumption with the implementation of a network using long-range wireless radio communication.

Despite remote monitoring strategies, the other strategy employs transformations to the data to improve its transmission performance. Pre-processing solutions offer some intelligence locally to process the data and extract higher levels of information. For instance, in [76], the authors proposed a three-tier structure for a real-time epileptic seizure detection system that contains a mobile device placed in the middle layer responsible for filtering, pre-processing, and extracting the electroencephalogram (EEG) characteristics. Filtering and aggregation strategies combine multiple samples of data to avoid sending redundant information. In this scope, the authors in [78] presented an aggregation scheme of lightweight privacy-preserving data. The researchers performed several experiments showing that by applying new filters at the edge of the network, communication resources are saved, avoiding false data injection. In turn, compression, as the name suggests, consists of shrinking the amount of data to be transmitted. Employing compression strategies, the authors in [77] demonstrated a medical processing system, which is responsible for inter-device communication and interpreting many wireless protocols used in e-Health applications. The system has a gateway node able to process data locally by applying data compression or fusion, and it also offers customized filtering and local storage.

#### 5.2.2. Data Processing

Differently from data collection strategies, some studies present strategies that focus on methods to process collected data. Such studies aim at the processing level layer in the fog architecture in which data is analyzed before transmission to remote locations. In the literature, articles focusing on this issue range between different e-Health applications. However, most of them concentrate efforts to develop health monitoring systems [79,80,81,82]. In this context, the authors in [79] come up with a new architecture model to minimize dependency on cloud storage and analytics for e-Health remote monitoring. The model resulted in a better system responsiveness and a lower bandwidth requirement due to the shorter distance between the data acquisition and data processing modules. In turn, in [80], the authors presented a fog-based monitoring system focusing on the detection and prevention of mosquito-borne diseases. The goal of the system is to analyze the physical sensed data and diagnose/differentiate the several types of mosquito-borne diseases at an early stage. In [81], the authors presented a robust infrastructure for electrocardiogram (ECG) monitoring applications, where primary and backup servers responsible for processing data are placed at the edge of the network. They proposed a processing architecture for optimizing the placement of server nodes to reduce energy consumption and networking equipment. The authors in [82] suggested an innovative framework based on IPv6 as a means of mitigating the difficulty of medical applications requiring low response time through the implementation of resource scheduling techniques. Such techniques were developed in a three-layer architecture, where the physiological data is collected at the body sensing layer and transferred to the fog layer for real-time processing.

Although health monitoring catches the attention of many studies, there are also some studies that focus on different aspects. Fog nodes are composed of hundreds of fog devices that can handle storage and small computational processes. Nonetheless, reaching the resource capacity offered by the conventional cloud is still a very tough challenge. For that reason, efficient management of resources is essential for fog environment operation. In this scope, in [83], the authors proposed a dynamic resource management solution for fog environments. The main goal of their strategy is to verify parameters from the active IoT devices and then estimate the best amount of resources required by a particular node, consequently avoiding the waste of resources. In the context of AAL, the authors in [84] proposed an AAL fog architecture containing a gateway node in charge of processing speech data from patients with Parkinson’s disease. The node works as an interface that processes the raw data collected by a smartwatch and sends it to the cloud infrastructure. Finally, the authors in [71] presented a study of the IoT paradigm for e-Health applications, in which they focus on the Network of Things concept. They introduced a framework for describing such environments for designing and implementing IoT solutions in healthcare.

#### 5.2.3. Critical Data Analysis

A group of articles focuses on the critical data analysis class, which considers strategies in which the main focus is to analyze data from critical conditions. In this scope, the authors in [85] demonstrated concerns regarding the security of patients data. They designed a fog-enabled architecture for healthcare services with a focus on risk assessment and information sharing. Additionally, the cooperation with the fog device enhances the privacy of medical data due to the implementation of robust operations of cryptography. Differently, other articles vary their strategies between ECG monitoring [86] to activity recognition for e-Health services [87]. Related to the first topic, the authors in [86] proposed an IoT-based application architecture that benefits from fog to enhance the quality of service of medical systems for local and remote patients. Instead of replacing the cloud infrastructure, a fog gateway is placed at the edge of the network to collaborate with the cloud, sharing the weight of handling all information from biosensors. In turn, related to the second topic, in [87], the authors presented a blockchain fog computing scheme for human activity recognition regarding e-Health services. The use of local servers placed at the network border adds preliminary data filtering, which improves the performance of complex operations and provides faster responses to relevant events.

For fog architectures that collect sensitive data, maintaining performance and decreasing costs are a key point for such systems. Although energy is consumed during the sensing procedure, the majority of energy consumption is related to computational tasks and data transmission. In order to quantify the effect of a great volume of data in mobile applications, the study in [44] demonstrated a systematic method based on whether offloading to the fog is better than to the cloud. In their approach, two concepts of applications were studied regarding latency and energy utilization. In addition, they focused on data transmission over WiFi and 4G LTE networks, which are common for mobile devices such as smartphones. Also focusing on efficiency, the authors in [42] considered task scheduling as a way of offloading traffic from the network core by strategically allocating services among fog nodes while minimizing the cost of resources. A three-layer hierarchy system is able to manage available resources located at both cloud and fog nodes and provide the most appropriate scheduling for the workflow. Finally, in the same direction, the authors in [31] investigated a resource management fog computing strategy applied to medical cyber-physical systems. The authors focused on the challenging environment for these systems that suffer from transmission instabilities between medical devices and the cloud data center. The model presented helps to share the burden of offloading traffic from the core network by distributing tasks through base station association, where virtual machines are deployed.

#### 5.2.4. Real-Time Feedback

Fog computing may benefit applications that need to detect unwanted events in real-time and respond to that event quickly. The real-time feedback class encompasses solutions that aim at providing quick responses to critical situations in healthcare environments. Several articles target the most variate set of applications in that scope: (i) critical event detection [61,88]; (ii) warning systems [89]; (iii) security issues [49,90]; and (iv) breath support systems [91]; In the first topic, in [88], the authors implemented a real-time signal processing algorithm for fall detection, which is able to deliver information to caregivers. These algorithms are executed at the network’s border by fog servers, which collect and process all health information. Similarly, ref. [61] suggested a solution to cerebrovascular accident mitigation, where the authors created a real-time analytic system for monitoring falls caused by strokes. The fall detection allocates tasks among smart devices and the cloud in a collaborative way. In the scope of the second topic, the authors in [89] presented a remote medical monitoring system as a reliable and efficient IoT-based approach that combines both machine learning algorithms and automated management components to provide monitoring and notification services. The system is responsible for deciding whether the information goes to the cloud when processing power is needed or to the local gateway, helping in decision making.

Although IoT devices play an important role in delivering services more efficiently in the health environment, many open issues related to privacy and security are still not addressed in the literature. In this context, the authors of [90] conceived a protection framework for medical applications based on fog computing able to detect privacy leakages. The advantage of this method is the capability to analyze data closer to the source of information instead of forwarding it to the cloud for processing. In such a case, the detection and blockage of privacy leakages become a lot faster than the currently used models. Likewise, in [49], the authors defined a new scheme applying fog computing that addresses the distribution of digital certificates in the IoT environment. The new scheme approach ensures that the revoked certificates can be immediately sent to the fog nodes, mitigating the risk of accepting a revoked certificate. Finally, regarding the last topic, the authors of [91] analyzed the effects caused by the implementation of fog-to-cloud computing models in health services. To exemplify the scenario, they proposed a breath support system for patients with pulmonary problems.

#### 5.2.5. Discussion

**Figure 4:**
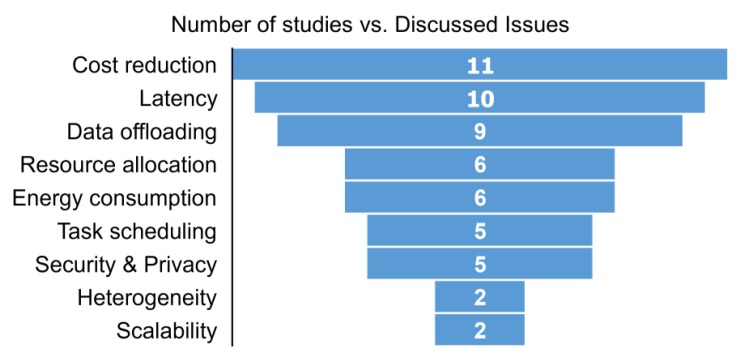
Amount of studies that focus on each issue.

Current studies employ concepts of fog computing for e-Health applications with different goals. Table 3 summarizes and classifies these research initiatives according to their main tasks, which are: (i) data collection [74,75,76,77,78]; (ii) data processing [71,79,80,81,82,83,84]; (iii) critical data analysis [31,42,44,85,86,87]; and (iv) real-time feedback [49,61,88,89,90,91]. Such functionalities in medical services focus on the enhancements of the security and privacy of sensitive information. The studies are concentrated in fog-based architectures to enable faster response times for real-time applications or resource scheduling techniques to minimize the total network bandwidth usage and consequently energy consumption. Data collection covers strategies that aim at acquiring data from health sensors quickly. In turn, data processing can be seen as the next level of data collection in which strategies focus on processing data acquired from health sensors. This process results in significant information that can be monitored in real-time. On the other hand, critical data analysis comprises studies focused on improving the healthcare processes by defining new architectures for such systems. More specifically, these solutions are concentrated on critical patients’ data from both the health and security points of view. Finally, real-time feedback is composed of solutions that monitor real-time data from patients focusing on diagnosing critical situations. In particular, this last group contains initiatives that provide real-time feedback for critical situations, helping physicians to improve response times for such situations. Figure 4 depicts the number of papers that focus on each one of these issues. The figure demonstrates that there is a high concentration of papers dealing with cost, latency, and data offloading. This indicates that most of the strategies are focused on improving healthcare services by focusing on data transfer but also by paying attention to costs reduction. Besides that, the figure also demonstrates that some studies focus on managing computational resources, which benefits not only the system performance but also energy efficiency. By improving energy efficiency it is possible to reduce costs at the same time.

### 5.3. RQ3: What Are the Current Open Issues Related to the Fog Computing on e-Health?

The fog computing approach plays an important role in healthcare by enabling applications to have fast data processing with low latency. Additionally, it addresses the security and confidentiality issues required by medical systems. Additionally, as fog nodes are closer to their end-users, fog computing helps to reduce bandwidth usage by offloading data traffic from the network core. In terms of e-Health systems, the concept of fog computing enhances data security and privacy, since all information is handled by fog devices and kept within the local network. It also helps to minimize medical expenses by reducing the amount of data exchanged between a particular health application and the cloud infrastructure. In addition, as the resources are placed closer to end-users, fog computing is able to provide instant responses for applications requiring real-time control. Although fog computing demonstrates better performance, it cannot totally replace cloud computing. Nonetheless, fog and cloud architectures will coexist while keeping their own advantages.

#### 5.3.1. Fog versus Cloud

Apart from the similarity between fog and cloud computing, there are many differences between the two architecture models. Applications hosted in the cloud are scalable and cheaper than in the fog model, due to the cloud’s huge storage capacity and hardware abstraction. Such abstraction is hard to be achieved in a fog network, since the edge devices must be acquired by the network owner. As a result of the heterogeneous nature of fog networks, a particular resource cannot be guaranteed in all fog devices [92]. Thus, keeping the system reliable requires a lot more effort, regarding complexity and cost, in fog when compared to the traditional cloud model. Ensuring a minimum of privacy and security in the fog model is a lot harder than in the cloud, since fog devices are normally maintained by many different service providers [93]. A single breach in a particular fog node may cause the system to operate incorrectly. In terms of connectivity in the fog environment, despite having hardware in full operation, if a single node loses access to the network, the entire system may be put at risk. Because fog is a decentralized model, ordinary hardware checks are much more complex and costly than in cloud infrastructures.

Nowadays, with the adoption of the IoT paradigm, current architectural approaches are unsustainable to provide services to the massive number of applications being developed. Many IoT devices are facing challenges related to latency, network bandwidth, dependability, privacy, and security, which cannot be handled in a cloud computing model. As a result, a fog computing approach has been presented as a propitious architecture to tackle these issues. The concept of fog computing is an extension of the cloud computing model to the network border. Both of them provide computational resources, storage, and network services to their end-users. Nonetheless, the fog paradigm differs from the cloud model in terms of network type, computing capacity, storage, physical location, and so on. As a new architecture, fog computing suggests several features that make it more complex than cloud [94]. According to [95,96,97,98], a comparison between these two models is presented in Table 4.

#### 5.3.2. Trends, Future Directions, and Open Issues

The emergence of the fog computing paradigm is relatively new when comparing to other well-established technologies such as cloud and IoT. Therefore, there is enough space for future developments and the integration of this paradigm into the e-Health scenario and other technologies. Some of the challenges faced by e-Health systems that can be addressed by fog computing can be classified in three groups of interest: (i) latency; (ii) power consumption; and (iii) heterogeneity and interoperability.

As some applications are commonly sensitive to delay, fog networks must guarantee that the response time of a certain request is within a limit. In other words, the fog system must employ mechanisms to verify if a specific task can be concluded or not regarding its defined metrics. In case a fog device is not able to deliver the service based on that metric, the service request must be rejected or forwarded to the cloud. With that in mind, defining a proper task allocation is of extreme importance. This scope encompasses a variety of application fields that can benefit from delay improvements, such as medical cyber-physical systems, ultra low latency applications, and tactile internet. In medical cyber-physical systems, devices monitoring physical parameters from both the environment and patients are integrated through the network [99]. Traditionally, medical devices produce data that must be transmitted to remote stations that process these data (for instance in the cloud). In such environments, the transmission of sensitive data among nodes with proper delay is crucial to improve the quality of medical services. From the ultra-low latency applications point of view, there are almost no initiatives that focus on applications that require constant low delay communication [100]. In such a context, ultra-low-latency networks are optimized to process a very high volume of data with a low tolerance for delay. These networks are designed to support real-time applications and react quickly to changes in the data streams. In addition, recent studies are focusing on the new tactile internet paradigm [101,102]. The goal is to bring the internet to a lower level of granularity: the human senses, such as touch. This will produce a new level of data generation that might scale rapidly. Therefore, collecting and transmitting this amount of data may be challenging, requiring communication infrastructures capable of handling it with low delay.

Unlike cloud systems, fog networks are composed of several decentralized fog devices commonly connected by battery or through inefficient communication interfaces. Defining a more efficient protocol to deal with resource allocation within the fog network may help to minimize the energy consumption in the fog environment. In that context, WSNs environments are promising, since in such architectures, the devices are characterized by low energy consumption [103]. WSNs are composed of a set of small nodes that employ low-energy protocols for communication and data exchange. In particular, combining fog and WSN has been explored by some researches in the literature; however there is still space for developing new strategies in this context. In the context of heterogeneity and interoperability, systems must inter-operate among several types of devices and vendors. Standards that ensure interoperability among such devices have not yet been addressed in the literature. For instance, it is possible to explore web services interfaces to allow data exchange between different modules and systems [104]. REST APIs (application programming interfaces) [105] can be used as a way of providing data for different systems in fog environments. Additionally, the development of new techniques for the announcements of available resources among fog devices as well as their communication protocols is fundamental to enable heterogeneous network interconnection.

## 6. Conclusions

In the present study, a comprehensive review of the fog computing framework was conducted, highlighting its major characteristics, such as its main architecture model, used network technologies, platforms for development, simulation tools, and its main security issues. To achieve this, a systematic literature review was performed that resulted in 48 articles analyzed carefully in this paper. Several application deployment cases regarding data collection and analysis were introduced before explaining the fog computing approach in the context of healthcare. Fog computing can be analyzed in the context of healthcare from two different points of view. The first one regards the fog infrastructure itself. Currently, studies agree that fog is a new layer between sensor devices and traditional cloud infrastructure. Such a model comprises some specific communication protocols and also several communication technologies, ranging from WPANs to LTE channels. This demonstrates that this new paradigm is used as an extension of current platforms using current technologies. The other point of view is the e-Health landscape, which comprises the use of computational applications to provide better healthcare services. Current studies focus on several issues that can affect the performance of systems and applications. In fact, one of the main issues these strategies target is cost and latency. On the one hand, cost reduction is one issue that is targeted in several areas and this is no different in healthcare scenarios. On the other hand, latency has a direct relation with time, which is a very important parameter in healthcare. Studies propose monitoring systems for data collection and processing that generate valuable feedback regarding patients’ health parameters. Fog computing can improve the time it takes to reach these results, since its hardware infrastructure is closer to the users.

The main contribution of the current research has two points of view. From the technical point of view, the article presented the main characteristics of fog computing architectures focused on the health scenario. In addition, the study defined the main tasks applications perform when employing such solutions. From the society point of view, the study demonstrated some possible paths for study combining fog and healthcare. More specifically, it demonstrated how future research might explore this field to provide solutions for healthcare applications in order to increase patients’ safety and security. Finally, considering future research, the topics include applying the research methodology from this survey to different paper databases. In the future, it is also possible to consider different areas and compare how fog computing is employed in each one of them. Additionally, the current research focused specifically on e-Health applications.

## Figures and Tables

**Table 1:** Summary of surveys and reviews related to the current research. IoT: Internet of things; WSN: wireless sensor networks.

Paper	Year	Technologies	Range	Taxonomy	Description
[18]	2017	Fog, IoT, WSN	2005–2016	-	Systematic review of wireless sensor networks applications that might benefit from fog computing in healthcare.
[16]	2018	Fog	2015–2018	-	Review classifying articles according to applications, diseases, research, characteristics, motivations, and challenges.
[17]	2018	Fog	2010–2018	-	Systematic review of fog computing issues for the applications domain in the healthcare scenario.
[19]	2019	Fog, Edge	-	-	Description of the edge and fog concepts, and proposal of an architecture that combines them for healthcare applications.
[15]	2019	Fog, IoT	2007–2017	🗸	Review of methods, systems, and surveys on IoT-based healthcare applications.
[36]	2020	Fog, IoT	2014–2018	🗸	Systematic literature review of resource management strategies in fog environments.
[20]	2020	Fog	2016–2019	🗸	Systematic literature review of fog computing applications for smart homes.

**Table 2:** Research questions (RQs).

ID	Question
RQ1	How do e-Health applications benefit from the fog computing architecture?
RQ2	What is the focus of e-Health systems employing the concept of fog computing?
RQ3	What are the current issues related to fog computing on e-Health?

**Table 3:** Reviewed e-Health applications.

	Reference Number	Application	Application Requirement								
			Resource Allocation	Task Scheduling	Heterogeneity	Scalability	Cost Reduction	Latency	Data Offloading	Security & Privacy	Energy Consumption
**Data Collection**	[74]	COPD monitoring system						🗸	🗸		
	[75]	Health monitoring system based on LoRa							🗸		🗸
	[76]	ECG monitoring system					🗸	🗸			
	[77]	Smart e-Health gateway			🗸	🗸					🗸
	[78]	Enhanced aggregation privacy scheme					🗸			🗸	
**Data Processing**	[82]	IPv6-based framework for e-Health applications			🗸		🗸	🗸			
	[84]	Parkinson speech device	🗸						🗸		
	[71]	Proposed framework for healthcare						🗸	🗸		
	[83]	Resource management implementation	🗸				🗸				
	[79]	Novel e-Health architecture for edge-IoT ecosystem						🗸	🗸		
	[80]	Fog-based system for mosquito diseases	🗸				🗸				
	[81]	Infrastructure for health monitoring applications									🗸
**Critical Data Analysis**	[86]	Proposed architecture for Enhanced QoS		🗸			🗸		🗸		
	[44]	Data offloading method						🗸	🗸		🗸
	[42]	Service allocation strategy		🗸			🗸		🗸		
	[31]	Concept of fog in medical system		🗸					🗸		
	[87]	Block-chain fog computing monitoring framework	🗸				🗸	🗸			
	[85]	Fog-assisted data sharing scheme					🗸			🗸	🗸
**Real-Time Feedback**	[88]	Real-time fall detection system						🗸			
	[61]	Fall monitoring system	🗸			🗸		🗸			
	[89]	Remote medical monitoring application	🗸	🗸			🗸				
	[91]	Breath support system					🗸			🗸	
	[49]	New security approach for fog nodes		🗸						🗸	
	[90]	Privacy leakage method for medical system						🗸		🗸	🗸

**Table 4:** Fog computing vs. cloud computing.

Requirements	Fog Computing	Cloud Computing
Geographical distribution	Distributed	Centralised
Location of server	At the edge of network	Within the Internet
Distance between client and server	Close (one hop)	Far (several hops)
Coverage	Extensive	Global
Latency	Low	High
Bandwidth	Low	High
Response time	short	long
Hardware	Limited resources	Scalable resources
Data storage	Temporary	Permanent
Flexibility	High	Limited
